# Differences in Brain Atrophy Pattern between People with Multiple Sclerosis and Systemic Diseases with Central Nervous System Involvement Based on Two-Dimensional Linear Measures

**DOI:** 10.3390/jcm13020333

**Published:** 2024-01-06

**Authors:** Małgorzata Siger, Jacek Wydra, Paula Wildner, Marek Podyma, Tomasz Puzio, Katarzyna Matera, Mariusz Stasiołek, Mariola Świderek-Matysiak

**Affiliations:** 1 Department of Neurology, Medical University of Lodz, Kopcinskiego Street 22, 90-414 Lodz, Poland; malgorzata.siger@umed.lodz.pl (M.S.); paula.wildner@gmail.com (P.W.); mariola.swiderek-matysiak@umed.lodz.pl (M.Ś.-M.); 2 Pixel Technology LLC, Piekna 1, 93-558 Lodz, Poland; j.wydra@pixel.com.pl (J.W.); m.podyma@pixel.com.pl (M.P.); t.puzio@pixel.com.pl (T.P.); k.matera@pixel.com.pl (K.M.)

**Keywords:** multiple sclerosis, systemic diseases, linear measures, brain atrophy, magnetic resonance imaging

## Abstract

Conventional brain magnetic resonance imaging (MRI) in systemic diseases with central nervous system involvement (SDCNS) may imitate MRI findings of multiple sclerosis (MS). In order to better describe the MRI characteristics of these conditions, in our study we assessed brain volume parameters in MS (n = 58) and SDCNS (n = 41) patients using two-dimensional linear measurements (2DLMs): bicaudate ratio (BCR), corpus callosum index (CCI) and width of third ventricle (W3V). In SDCNS patients, all 2DLMs were affected by age (CCI *p* = 0.005, BCR *p* < 0.001, W3V *p* < 0.001, respectively), whereas in MS patients only BCR and W3V were (*p* = 0.001 and *p* = 0.015, respectively). Contrary to SDCNS, in the MS cohort BCR and W3V were associated with T1 lesion volume (T1LV) (*p* = 0.020, *p* = 0.009, respectively) and T2 lesion volume (T2LV) (*p* = 0.015, *p* = 0.009, respectively). CCI was associated with T1LV in the MS cohort only (*p* = 0.015). Moreover, BCR was significantly higher in the SDCNS group (*p* = 0.01) and CCI was significantly lower in MS patients (*p* = 0.01). The best predictive model to distinguish MS and SDCNS encompassed gender, BCR and T2LV as the explanatory variables (sensitivity 0.91; specificity 0.68; AUC 0.86). Implementation of 2DLMs in the brain MRI analysis of MS and SDCNS patients allowed for the identification of diverse patterns of local brain atrophy in these clinical conditions.

## 1. Introduction

The accessibility and effectiveness of disease-modifying therapy (DMT) in multiple sclerosis (MS) have been constantly increasing in recent years. The current revision of the MS diagnostic criteria (McDonald 2017) [1] facilitated the diagnostic process; however, high percentages of misdiagnoses have been reported in both North American and European populations [2,3,4]. In systemic diseases with central nervous system involvement (SDCNS), clinical presentation and findings of magnetic resonance imaging (MRI) may be similar to those of MS. Our recently published review [5] summarized the latest data describing similarities and differences between MS and SDCNS, including radiological characteristics. Although MRI is one of the most important paraclinical tools in the diagnosis of MS, its specificity is not satisfactory [6]. Focal white matter lesions similar to those observed in MS have been described in many other neurological disorders including SDCNS. Gadolinium-enhancing T1 lesions and T2 hyperintense lesions on MRI correspond in vivo with inflammatory reactions and focal demyelination, but these findings do not cover the whole spectrum of MS pathology and in particular fail to demonstrate the extent of neurodegenerative processes [6,7]. Brain and spinal cord atrophy are considered as hallmarks of neurodegeneration in MS [8,9,10]. Both white and grey matter volume loss as well as local atrophy of particular brain structures (e.g., thalamus) have been repeatedly demonstrated as strong correlates of physical and cognitive disability progression in MS [11,12,13,14]. However, little is known about brain atrophy in SDCNS patients. Data published until now in patients with systemic lupus erythematosus (SLE) with CNS involvement (CNSI) showed a correlation between disease duration, activity and caudate and fronto-temporal atrophy [15,16]. In vivo brain atrophy is usually estimated by measurement of brain volume (BV) change [8,17,18]. Assessment of brain atrophy on MRI with the use of two-dimensional linear measurements (2DLMs), including bicaudate ratio (BCR), corpus callosum index (CCI) and width of third ventricle (W3V), has been proposed as an easy-to-use alternative in daily clinical routine [19,20,21]. BCR is suggested as a marker of deep frontal subcortical white matter atrophy, CCI is an accepted measurement of local white matter atrophy and W3V reflects central brain atrophy [19,20,21]. Recently published results indicate that 2DLMs applied in clinical practice may be useful as markers of long-term clinical progression and may correlate with neurological and cognitive impairment in MS patients [19,20,21]. Taking into consideration the above-mentioned limitations of conventional MRI, in our work we applied 2DMLs in order to characterize brain atrophy patterns in a group of newly diagnosed MS and SDCNS patients.

## 2. Materials and Methods

This cross-sectional, prospective study was conducted as a part of a larger, multiparametric study investigating the clinical, molecular and imaging characteristics of people undergoing differential diagnosis because of focal brain lesions detected on MRI [22]. The participants were recruited consecutively during or directly after the diagnostic process between July 2018 and October 2019 in the Department of Neurology and Neurology Outpatient Clinic, Medical University of Lodz, Poland. The participants were included in one of two groups: group 1—MS—or group 2—SDCNS. Inclusion criteria were as follows: Group 1: diagnosis of MS based on McDonald criteria 2017; relapsing–remitting course of MS; no disease exacerbation and/or steroid treatment within at least 3 months before enrolment into the study; no history of any disease-modified treatment. Group 2: diagnosis of SDCNS confirmed based on clinical criteria laboratory tests (including peripheral blood and cerebro-spinal fluid examination) and radiological assessment; no history of steroid or other disease-modified treatment. Patients from both groups had to be 18–55 years old and have brain MRI lesions indicating a demyelinating process suggestive of MS. Exclusion criteria for all participants included contraindication to MRI and diagnosis of diseases other than MS/SDCNS affecting CNS including infectious, metabolic and other neurologic disorders. Demographic and medical data were collected in a local medical database. The level of physical disability in MS patients was assessed with the Expanded Disability Status Scale (EDSS) [23]. 

The study was conducted according to the guidelines of the Declaration of Helsinki (1964) and its later amendments and received the approval of the Local Ethics Committee of the Medical University of Lodz (approval number/360/17/KE, 21 November 2017, RNN/231/18KE, 12 June 2018). All subjects gave their informed consent before participating in the study.

### 2.1. MRI Acquisition

All participants underwent brain MRI examinations performed on a 3.0 T scanner (Vida, Siemens, Munich, Germany) with a 20-channel head coil. The acquisition protocol was set according to the guidelines of the Polish Neurological Society and Polish Medical Society of Radiology [24]. The MRI protocol included the following sequences: a high- resolution axial 3-dimensional (3D) T1-weighted magnetization-prepared rapid gradient- echo (MPRAGE) (repetition time (TR) = 2200 ms; echo time (TE) = 2.46 ms; inversion time (TI) = 900 ms; field of view (FOV) = 256; number of slices = 167; pixel size = 1 × 1 × 1 mm), sagittal isotropic 3D T2-weighted fluid-attenuated inversion recovery (FLAIR) (TR = 2560 ms; TE = 135 ms; TI = 6700 ms; FOV = 256; number of slices = 192), proton density PD/T_2_-weighted (TR = 2560 ms; TE1/TE2 = 90/30 ms; FOV = 256; number of slices = 46; slice thickness = 3.0 mm) and 3D T_1_-MPARAGE (repetition time (TR) = 2200 ms; echo time (TE) = 2.46 ms; inversion time (TI) = 900 ms; field of view (FOV) = 256; number of slices = 167; pixel size = 1 × 1 × 1 mm) after intravenous contrast administration (gadolinium-based, gadobutrol 0.1 mmol/kg body weight).

### 2.2. MRI Postprocessing

**Figure 1:**
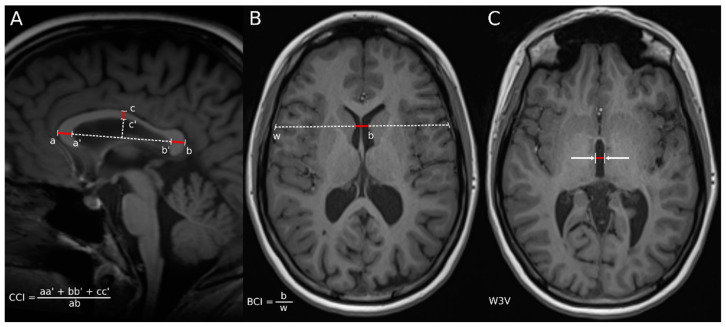
Two-dimensional linear measurements of brain atrophy: (**A**) corpus callosum index (CCI), (**B**) bicaudate ratio (BCR), (**C**) width of third ventricle (W3V). (**A**) CCI was calculated by drawing the greatest anteroposterior and craniocaudal axes at their midpoint, leading to the points a, a’, b, b’ and c, c’. The anterior, medium and posterior segments of the CC were then measured and normalized by the greatest anteroposterior diameter (segment ab). (**B**) Measurement of BCR was defined as the minimum intercaudate distance (red line “b” divided by brain width along the same line (dotted line “w”). (**C**) MR image in transverse plane showing measurement of third ventricle width (red line).

MRI lesion analysis (T2-lesion volume on T2/FLAIR image (T2LV) and T1-lesion volume (T1LV)) was performed using a semi-automated, edge-finding tool in Jim software package (v7.0, Xinapse Systems Ltd. West Bergholt, Essex, CO6 3BW, UK, http://www.xinapse.com (accessed on 1 November 2023)) which is well established and widely used throughout the neuroimaging MS research community. MRI lesion analysis was performed by one of the co-authors (MS), with >20 years of experience in MRI analysis. All 2DLMs were assessed using the automatic Exhibeon (v.3) software (https://www.allerad.com/en/dicom-viewer, accessed on 1 May 2020), Pixel Technology Gmbh). Details of the Exhibeon pipeline have been previously reported elsewhere [13]. The CCI was measured based on the method described by Figueira et al. [25]. The measurement was performed on a mid-sagittal MPRAGE image, where the septum pellucidum was visible and all elements of the corpus callosum (CC) were well visualized (Figure 1A). The measurement was assessed by drawing the greatest anteroposterior and craniocaudal axes at their midpoint, leading to the points a, a’, b, b’ and c, c’. The anterior, medium and posterior segments of the CC were then measured and normalized using the greatest anteroposterior diameter. The final CCI was obtained according to the formula described elsewhere [25,26,27]. BCR was measured based on the method described by Bermel et al. [28] in the axial section of MPRAGE images where the frontal horns were clearly visible and the septum was the thinnest. The BCR was the minimum intercaudate distance divided by brain width along the same line (Figure 1B). W3V was assessed on 3D T1-weighted axial images based on the methods proposed by Bermel et al. [29]. The location where the third ventricle (3V) reaches its most optimal width was initially assessed (a slice between the adjacent slices where expansion or contraction of the ventricle was observed had been selected). The center of the 3V was then determined by measuring its length in the AP (anteroposterior) direction, and a linear measurement of the ventricle width in the RL (right–left) direction was performed at the determined location (Figure 1C).

CCI, BCR and W3V were assessed by two independent researchers: a neuroradiologist (TP with 8 years of experience in MRI) and an image analyst (KM with 7 years of experiences in MRI analysis) blinded to clinical diagnosis. Inter-rater reliability was measured with the intraclass correlation coefficient (ICC). The inter-rater ICCs for the two raters were 0.93, 0.94 and 0.97 for BCR, CCI and W3V, respectively.

### 2.3. Statistical Analysis

Statistical analysis was performed with Python 3.8 (www.python.org (accessed on 1 November 2023)) programming language, with the statistical libraries scikit-learn 1.0.2 (www.scikit-learn.org (accessed on 1 November 2023)), statsmodels 0.13.1 (www.statsmodels.org (accessed on 1 November 2023)) and optuna 2.10.0 (www.optuna.org (accessed on 1 November 2023)). The distribution of each variable was tested with the d’Agostino–Pearson normality test. The equality of means in subgroups was assessed with the t-Student test for all normally distributed variables and with the Mann–Whitney U test for the others. As gender is a categorical variable, the equality of its distribution between MS and SDCNS patients was tested with the chi-squared test. *p* values of <0.05 were considered statistically significant. Inter-rater agreement analysis was conducted using interclass correlation coefficients (ICCs). Based on statistical recommendation [30], ICC values of <0.40 were considered poor, 0.40–0.75 fair to good and >0.75 excellent. The impact of the demographics, disease duration, T1LV and T2LV on 2DLMs was determined by the slope of the regression line (β). For each of the 2DLMs, we estimated a series of univariate linear regression models containing one of the mentioned independent variables. The values of the coefficients corresponding to explanatory variables indicated the strength of the relation and the *p*-value implied whether the given relation is statistically significant. Stepwise logistic regression models and ROC curve analysis including demographic, clinical and MRI volumetric parameters were used to describe MS and SDCNS. We used the repeated k-fold cross-validation method to search for the best model [31,32]. As the independent variables in the best model selection procedure, we included all the 2DLMs, age, gender, disease duration, T1LV and T2LV.

## 3. Results

### 3.1. Patients

This was a prospective cohort study of 99 patients: 58 with MS (male/female = 17/41) and 41 with SDCNS (male/female = 5/36). In total, 14 patients in the SDCNS group were diagnosed with systemic lupus erythematosus (SLE), 12 with undifferentiated connective tissue disease, 9 with CNS vasculitis, 3 with Sjögren’s syndrome and 3 with neurosarcoidosis. Patients with SDCNS were significantly older than patients with MS (*p* = 0.001). There were no differences in gender distribution and disease duration between the MS and SDCNS groups. Demographic and clinical details of the MS and SDCNS patients are presented in Table 1.

### 3.2. MRI Results

Differences in the association of demographic factors and/or lesion volumes in brain MRI with 2DLMs were found between MS and SDCNS patients (Table 2). In SDCNS patients, all 2DLMs were significantly driven by age. Lower CCI as well as higher BCR and W3V were associated with older age in SDCNS patients (*p* = 0.005, β = −0.0017; *p* < 0.001, β = 0.0012; *p* < 0.001, β = 0.1090, respectively). In MS patients, higher BCR and higher W3V correlated with older age (*p* = 0.001, β = 0.0009; *p* = 0.015, β = 0.0576, respectively) but we did not observe an association of age with CCI (*p* = 0.768, β = 0.0002) (Table 2). Regarding radiological parameters, 2DLMs were driven by T1LV and T2LV in MS patients only. Lower CCI was associated with higher T1LV (*p* = 0.015, β = −0.0165) (Table 2). Moreover, higher BCR and higher W3V were associated with higher T1LV and T2LV (BCR: *p* = 0.020, β = 0.0076; *p* = 0.015, β = 0.0112; W3V: *p* = 0.009, β = 0.7467; *p* = 0.009, β = 1.0467, respectively) (Table 2). Gender and disease duration had no impact on 2DLMs in either the MS or SDCNS patients (Table 2). 

After adjusting for age, we have also found differences in BCR and CCI between the MS and SDCNS groups (Table 3). BCR was significantly higher in SDCNS compared with MS patients (*p* = 0.01) and CCI was significantly lower in MS compared with SDCNS patients (*p* = 0.01). W3V was comparable between the MS and SDCNS groups (*p* = 0.16) (Table 3). Volumetric analysis of focal lesions showed that T2LV and T1LV in the MS patients were significantly higher than in the SDCNS patients (*p* < 0.001) (Table 3). 

**Figure 2:**
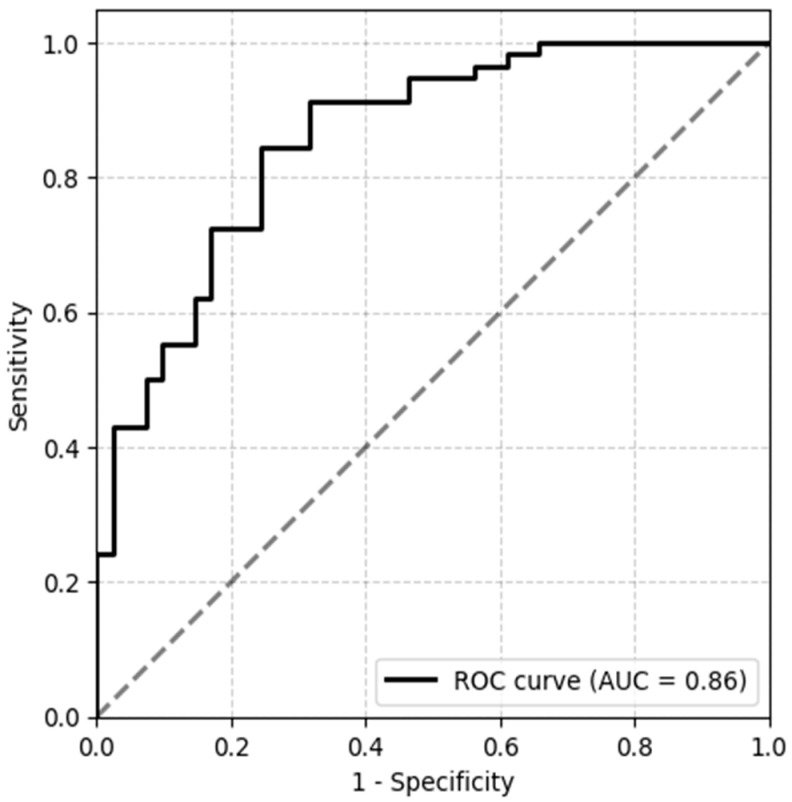
Receiver Operating Characteristic (ROC) curve analysis based on the best predictive model discriminating MS and SDCNS patients, including gender, BCR and T2LV as the explanatory variables.

In the final step of the analysis, we assessed a combination of demographic, clinical and MRI variables in the MS and SDCNS patients in order to characterize the best set of parameters discriminating both groups. We identified gender, BCR and T2LV as the explanatory variables in the best predictive model to distinguish MS and SDCNS patients (sensitivity 0.91, specificity 0.68, AUC 0.86), where female gender, higher BCR and lower T2LV predisposed patients to a diagnosis of SDCNS. The ROC curve of the best predictive model is presented on Figure 2.

## 4. Discussion

The application of 2DLMs to estimate the brain volume loss in MS has been investigated in numerous studies in correlation with clinical presentation, cognitive impairment and MRI focal white matter lesions [19,20,28,33,34,35,36,37,38]. Brain atrophy parameters were also assessed in SLE patients with CNSI [15,16,39,40,41,42,43]. However, to the best of our knowledge, there are no published data describing the use of 2DLMs in order to compare brain atrophy in SDCNS and MS patients. 

In our analysis, we demonstrated the different impact of demographic parameters and lesion volume on 2DLMs in MS and SDCNS patients. One of the investigated 2DLMs, CCI, a parameter assessing CC atrophy, was incorporated in multiple studies on MS [20,26,35,44]. CC atrophy was also explored in patients with SLE and primary Sjögren’s syndrome with CNSI [15,39,40,45]. In our study, we found that in contrast to SDCNS, in MS patients CCI was not driven by age. Our results remain in line with the findings of another study indicating that, in MS, CC atrophy represents an age-independent process [15]. The differences observed in the current study can be interpreted as a result of diverse pathological mechanisms underlying CC atrophy in particular diseases. In the SDCNS group, CC degeneration seems to be more dependent on age-related changes, whereas in MS the effect of age could be potentially neutralized by the extent of early disease-specific degenerative processes in CC—an anatomical structure known to be highly affected in the disease [25,26,27,46,47]. This assumption may remain in concordance with the observation that other 2DLMs (BCR and W3V) correlated with age in both the MS and SDCNS patients. BCR was applied as brain atrophy parameter in previous studies in MS [28,33,34,35], and correlation between BCR and age of patients was reported [19,28,44,48]. Additionally, in a recent study by Nishizawa et al., a BCR cut-off value of 0.1627909 was suggested for discriminating between MS patients with mild and severe total brain and grey matter volume loss, physical disability and cognitive impairment [44]. However, there are no data concerning the impact of age on BCR in SDCNS patients. Thus, based on the accepted interpretation that BCR reflects deep frontal subcortical white matter atrophy [28,33,34,35,49], our findings highlight a relationship between age and this process not only in MS but also in SDCNS patients. Another 2DLM commonly used to assess brain atrophy is 3VW. Enlargement of the 3V has been well documented in patients with MS [19,20,33,34,35,50,51]. This widening of the 3V is suggested to be a marker of central brain atrophy [50]. In this regard, in our study we found that both in MS and in SDCNS patients, W3V was driven by age. Our results are in agreement with findings described earlier by Kalinowska-Lyszczarz et al. regarding MS patients, but are still in contrast with data from the SLE-CNSI group, where an age-dependent enlargement of 3V in the SLE-CNSI group was not observed [15]. This discrepancy may be associated with the relative heterogeneity of our SDCNS group, which makes a direct comparison with the group investigated by Kalinowska-Lyszczarz et al. [15] difficult. 

We also observed that T1LV and T2LV had different correlations with 2DLMs in MS and SDCNS patients. CCI was driven by T1LV only in the MS group, which stays in line with previous studies reporting correlations between T1LV and CCI/CC area [52,53]. We may speculate that in MS, CC white matter atrophy expressed as CCI and focal brain tissue destruction expressed as T1LV represent inter-related processes, whereas, as suggested above, in SDCNS, neurodegenerative processes employ other mechanisms. In contrast to the SDCNS patients, in the MS group the higher BCR was driven by higher T1LV and T2LV. These results are supported by similar data obtained earlier by Bermel et al. [28]. Additionally, 3V enlargement was also associated with higher T1LV and T2LV only in the MS group. Again, these observations might suggest that deep frontal subcortical white matter atrophy (measured by BCR) as well as central brain atrophy (represented by 3V enlargement) [28,33,34,35,49,50] are significantly dependent on inflammatory and/or degenerative processes represented by focal white matter pathology in MS but not in SDCNS patients.

The next important finding of our study is the differences in BCR and CCI between MS and SDCNS groups. BCR was significantly higher in SDCNS than in MS patients. Taking into account BCR interpretation as described above, this finding indicates that the atrophy of deep frontal subcortical white matter is more pronounced in SDCNS than in MS patients with the same disease duration. Such observation supports the assumption that neurodegenerative processes in these clinical conditions are driven by divergent pathological pathways. However, this area definitely needs to be further investigated with more advanced diagnostic tools allowing for molecular assessment of myelin and/or axonal pathology [54,55]. We also found that CCI was significantly lower in MS compared with SDCNS patients. Earlier studies showed that CCV is significantly lower in SLE-CNSI and SLE patients without CNS involvement than in healthy individuals both in adult [15,39,40,56,57] and pediatric populations [43]. In the work described above [15], the authors showed that the volume of the posterior part of CC is the best volumetric predictor distinguishing MS from SLE-CNSI patients. In our study, we measured CCV in MS and SDCNS patients using a CCI—a method potentially applicable in the real-world clinical settings of an average MS center. The lower CCI values observed in our MS patients may indicate that, at the same disease duration, local white matter atrophy is more advanced in MS than in SDCND patients.

In our study, we also found significantly higher T2LV and T1LV in MS than in SDCNS patients. These results underscore focal white matter destruction as a process more pronounced in MS than in SDCNS patients beginning from the earliest phases of the disease, which is concordant with the understanding of CNS pathology in MS [5,14,58]. Additionally, this observation seems to support a less clear association of CNS symptoms with focal brain pathology in systemic diseases. Importantly, our findings corroborate the results of a previously published study which also detected differences in white matter lesion load between patients with MS and SLE-CNSI [15]. 

Finally, we found that the multiparametric model including gender, BCR and T2LV optimally identifies MS from SDCNS patients. The final predictive model was an ensemble of multiple parameters trained on different training and validation data splits (repeated k-fold cross-validation method). Based on the analysis of the constituent models, we found that female gender, higher BCR and lower T2LV may predispose patients to a diagnosis of SDCNS. Stepwise logistic regression models and ROC curve analysis including demographic, clinical and MRI volumetric parameters to differentiate MS and SLE-CNSI patients were also performed in a previously cited study [15]. In this research, the fourth ventricle volume, posterior corpus callosum volume and third ventricle ratio were the best predictors which distinguished MS from SLE-CNSI patients. However, the statistical models mentioned above did not include BCR, which was the best predictor of all of the 2DLMs assessed in our study. 

### Limitations

One potential limitation of our study was the heterogeneity of the SDCNS group and the relatively low number of subjects included in this group. This is a direct consequence of the fact that SDCNS diseases are uncommon and larger cohorts of patients are very difficult to follow. The second limitation is the lack of a healthy control group, but the main goal of our study was to compare 2DLMs in MS and SDCNS patients. Such a situation also reflects the typical conditions of real-world clinical practice, where MRI scans are not routinely performed in healthy subjects. Finally, another limitation is the unbalanced distribution of patient age within MS and SDCNS group, but this parameter was included as one of the co-factors in statistical analysis. 

## 5. Conclusions

Based on 2DLMs, we showed different patterns of local atrophy in newly diagnosed MS and SDCNS patients, with the same duration of disease. Most interestingly, our findings suggest that brain atrophy in these two different groups of patients is associated with distinct demographic and conventional MRI parameters. BCR combined with gender and T2LV was identified as the best set of variables distinguishing MS and SDCNS patients.

## Figures and Tables

**Table 1:** Demographic and clinical characteristics of the study population.

Characteristic	MS n = 58	SDCNS n = 41	*p*-Value
Age (years) mean	36.69	44.15	0.001 ^#^
Age (years) range (SD)	33.92–39.45 (10.43)	40.52–47.76 (11.33)	
Female-to-male ratio	17/41	5/36	0.08
Disease duration (years) mean (SD)	2.37 (2.74)	3.27 (5.86)	0.51
EDSS median	1.25 (1.00)	n/a	n/a

Abbreviations: MS—multiple sclerosis; SDCNS—systemic disease with central nervous system involvement; EDSS—Expanded Disability Status Scale; SD—standard deviation; n/a—not applicable; ^#^ statistically significant (*p* value < 0.05).

**Table 2:** The impact of demographic, clinical and radiological parameters on 2DLMs in MS and SDCNS patients.

Variable	Index	MS		SDCNS	
		β	*p*-Value	β	*p*-Value
age	CCI	0.0002	0.768	–0.0017	0.005 ^#^
	BCR	0.0009	0.001 ^#^	0.0012	<0.001 ^#^
	W3V	0.0576	0.015 ^#^	0.1090	<0.001 ^#^
gender	CCI	−0.0120	0.360	−0.0241	0.282
	BCR	−0.0099	0.116	0.0026	0.822
	W3V	−0.5127	0.353	1.0128	0.354
disease duration	CCI	0.0042	0.734	0.0002	0.985
	BCR	0.0089	0.133	–0.0008	0.899
	W3V	0.8982	0.081	–0.1638	0.778
T1LV	CCI	–0.0165	0.015 ^#^	0.0062	0.336
	BCR	0.0076	0.020 ^#^	–0.0027	0.414
	W3V	0.7467	0.009 ^#^	–0.2589	0.396
T2LV	CCI	–0.0188	0.050	–0.0026	0.729
	BCR	0.0112	0.015 ^#^	0.0046	0.212
	W3V	1.0467	0.009 ^#^	0.3882	0.271

Abbreviations: MS—multiple sclerosis; SDCNS—systemic disease with central nervous system involvement; T2LV—T2 lesion volume; T1LV—T1 lesion volume; BCR—bicaudate ratio; CCI—corpus callosum index; W3V—width of third ventricle; β—the slope of the regression line; ^#^ statistically significant (*p* value < 0.05).

**Table 3:** MRI parameters in MS and SDCNS patients.

Parameter/Groups	MS (n = 58)	SDCNS (n = 41)	*p*-Value
BCR mean, (SD)	0.09 (0.02)	0.10 (0.02)	0.01 ^#^
CCI mean, (SD)	0.39 (0.04)	0.47 (0.04)	0.01 ^#^
W3V mm mean, (SD)	1.73 (1.78)	2.34 (2.01)	0.16
T2LV mL (SD)	3.70 (5.73)	0.66 (0.97)	<0.001 ^#^
T1LV mL (SD)	1.77 (3.48)	0.14 (0.27)	<0.001 ^#^

Abbreviation: MS—multiple sclerosis; SDCNS—systemic disease with central nervous system involvement; T2LV—T2 lesion volume, ml (milliliter); SD—standard deviation; T1LV—T1 lesion volume, mL (mililiter); BCR—bicaudate ratio; CCI—corpus callosum index; W3V—width of third ventricle; ^#^ statistically significant (*p* value < 0.05).

## Data Availability

Data and materials are available in local database and in a standardized format.
